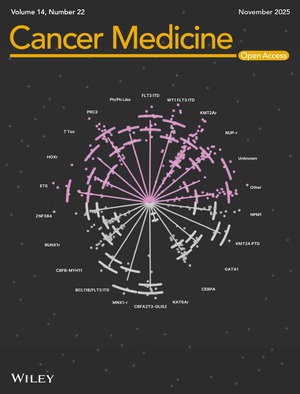# Cover Image

**DOI:** 10.1002/cam4.71389

**Published:** 2025-11-20

**Authors:** Quenton Rashawn Bubb, Elena Sotillo, Rebecca M. Richards, Crystal L. Mackall, Tanja A. Gruber, Agnieszka Czechowicz

## Abstract

The cover image is based on the article *Transcriptomic Diversity of Pediatric Acute Myeloid Leukemia Genetic Drivers Correlates With Clinical Outcome and Expression of Stemness‐Related Genes* by Quenton Rashawn Bubb et al., https://doi.org/10.1002/cam4.71325.